# Supplementary material for: Eicosapentaenoic Acid and Urolithin a Synergistically Mitigate Heat Stroke-Induced NLRP3 Inflammasome Activation in Microglial Cells
Source: Nutrients. 2025 Sep 25;17(19):3063. doi: 10.3390/nu17193063 (PMC12526280; doi:10.3390/nu17193063)
Supplement: Supplementary file 1 [file nutrients-17-03063-s001.zip › nutrients-3859713-supplementary.pdf]

Supplementary Table S1. Primer sequences for qRT-PCR.

Gene	Forward/Reverse	Sequence (5′-3′)
IL1β	Forward	TGGACCTTCCAGGATGAGGACA
	Reverse	GTTCATCTCGGAGCCTGTAGTG
Nlrp3	Forward	ATGCTGCTTCGACATCTCCT
	Reverse	AACCAATGCGAGATCCTGAC
Il-4	Forward	GGTCTCAACCCCCAGCTAGT
	Reverse	GCCGATGATCTCTCTCAAGTGAT
Il-6	Forward	CTGCAAGAGACTTCCATCCAGTT
	Reverse	AGGGAAGGCCGTGGTTGT
Il-8	Forward	GGTCTGCTACGGGCTCACA
	Reverse	CCCGGTGTTTCTGCCTCAT
Il-10	Forward	CGGGAAGACAATAACTGCACCC
	Reverse	CGGTTAGCAGTATGTTGTCCAGC
Il-13	Forward	CCTGGCTCTTGCTTGCCTT
	Reverse	GGTCTTGTGTGATGTTGCTCA
Il-16	Forward	CACGCAGACTTCATCCTCCACA
	Reverse	AGCTATAGTCCATCCGTGCCTG
Il-18	Forward	GACAGCCTGTGTTCGAGGAT
	Reverse	TGGATCCATTTCCTCAAAGG
Tnfα	Forward	GGTGCCTATGTCTCAGCCTCTT
	Reverse	GCCATAGAACTGATGAGAGGGAG
iNos	Forward	GCAGCTGGGCTGTACAAA
	Reverse	AGCGTTTCGGGATCTGAAT
Cox2	Forward	TGCATTCTTTGCCCAGCACT
	Reverse	AAAGGCGCAGTTTACGCTGT
Mcp1	Forward	GCTACAAGAGGATCACCAGCAG
	Reverse	GTCTGGACCCATTCCTTCTTGG
Hprt	Forward	CTGGTGAAAAGGACCTCTCGAAG
	Reverse	CCAGTTTCACTAATGACACAAACG

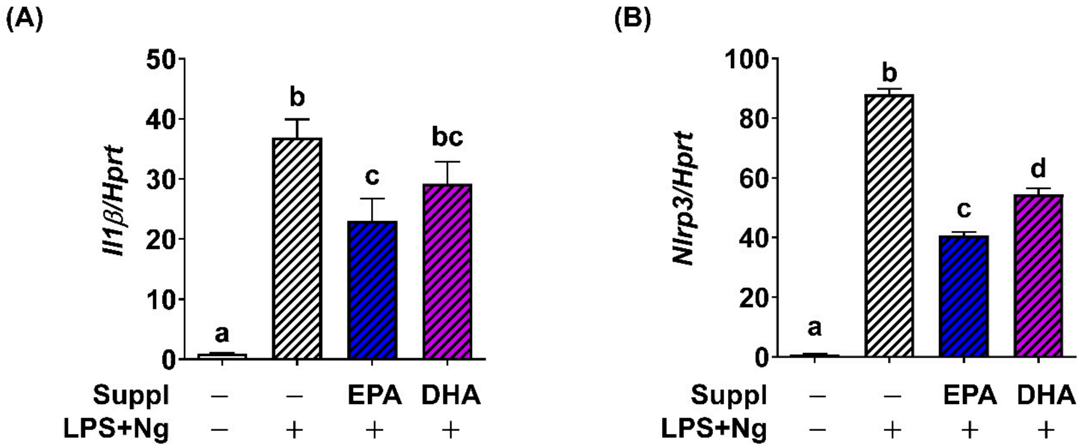

Supplementary Figure S1. EPA and DHA alleviate NLRP3 inflammasome related gene expression in BV2 cells. (**A**) *Il1β* and (**B**) *Nlrp3* gene expression, induced by inflammation activation upon LPS plus Ng treatment in BV2 cells in the presence of EPA or DHA. All data are shown as mean ± SEM. Values not sharing a common letter differ significantly ($p < .05$) by one-way ANOVA. LPS, lipopolysaccharide; Ng, nigericin; EPA, eicosapentaenoic acid; DHA, docosahexaenoic acid
